# A review of the genetic background in complicated *WT1*-related disorders

**DOI:** 10.1007/s10157-024-02539-x

**Published:** 2024-07-13

**Authors:** China Nagano, Kandai Nozu

**Affiliations:** Department of Pediatrics, Kobe University Graduate School of Medicine, 7-5-2 Kusunoki-Cho, Chuo-Ku, Kobe, 650-0017 Japan

**Keywords:** WT1, DNA binding, Denys-Drash syndrome, C2H2, Frasier syndrome

## Abstract

The Wilms tumor 1 (*WT1*) gene was first identified in 1990 as a strong candidate for conferring a predisposition to Wilms tumor. The WT1 protein has four zinc finger structures (DNA binding domain) at the C-terminus, which bind to transcriptional regulatory sequences on DNA, and acts as a transcription factor. WT1 is expressed during kidney development and regulates differentiation, and is also expressed in glomerular epithelial cells after birth to maintain the structure of podocytes. *WT1*-related disorders are a group of conditions associated with an aberrant or absent copy of the *WT1* gene. This group of conditions encompasses a wide phenotypic spectrum that includes Denys–Drash syndrome (DDS), Frasier syndrome (FS), Wilms–aniridia–genitourinary–mental retardation syndrome, and isolated manifestations of nephropathy or Wilms tumor. The genotype–phenotype correlation is becoming clearer: patients with missense variants in DNA binding sites including C2H2 sites manifest DDS and develop early-onset and rapidly developing end-stage kidney disease. A deeper understanding of the genotype–phenotype correlation has also been obtained in DDS, but no such correlation has been observed in FS. The incidence of Wilms tumor is higher in patients with DDS and exon-truncating variants than in those with non-truncating variants. Here, we briefly describe the genetic background of this highly complicated *WT1*-related disorders.

## Introduction

**Fig. 1:**
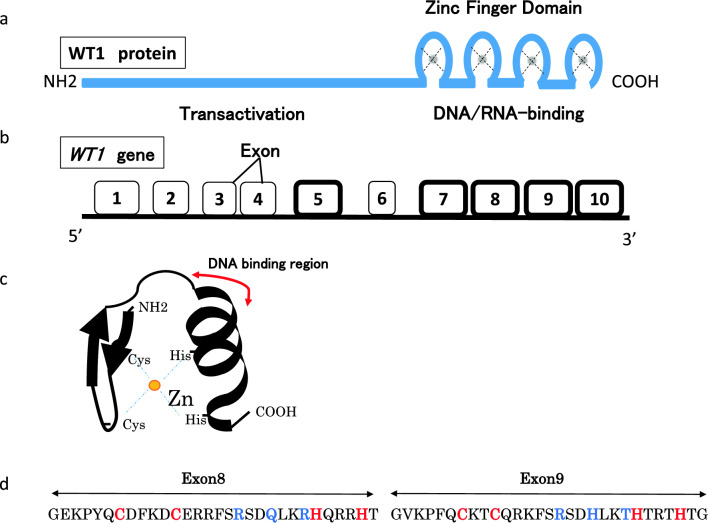
Schematic representations of the *WT1* gene and protein. (**a**) Known functional domains of the WT1 protein include DNA/RNA binding (zinc finger) domain. (**b**) *WT1* comprises 10 exons. The inclusion of exon 5 leads to the insertion of 17 amino acid residues into the regulatory domain of *WT1*. (**c**) C2H2-type zinc fingers contain a short beta hairpin and an alpha helix, where a single zinc atom is held in place by C2H2 residues in a tetrahedral array. (**d**) The sequence-recognition amino acids at the protein–DNA interface in exons 8 and 9 are shown in blue. The Cys2–His2 structural amino acids that coordinate the zinc ions and hydrophobic core are shown in red

**Fig. 2:**
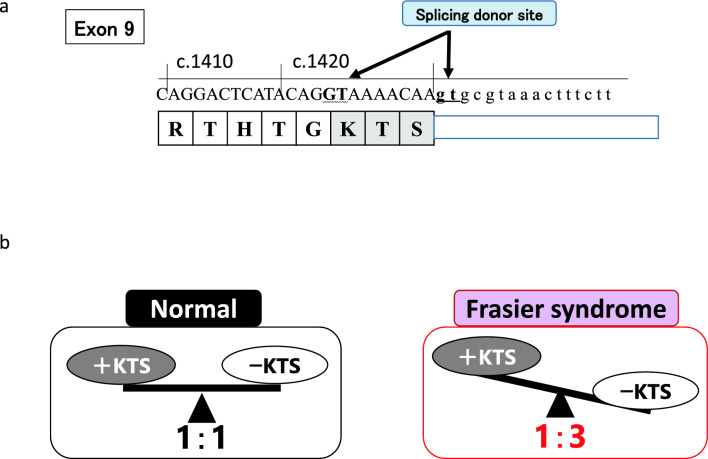
(**a**) Alternative splicing at the end of exon 9 produces the tripeptide KTS, which is inserted between zinc fingers 3 and 4. (**b**) Schematic representation of the balance of + KTS and − KTS isoforms in normal cases and its imbalance in Frasier syndrome

The *WT1* gene was isolated in 1990 as the causative gene of Wilms tumor and is located on chromosome 11p13 [1, 2]. The gene spans approximately 50 kb and contains 10 coding exons: exons 1–6 encode an N-terminus Gln/Pro-rich domain and exons 7–10 encode four consecutive (Cys)2-(His)2 zinc finger C-terminus domains (C2H2 site) (Fig. 1) [3]. This gene encodes a transcription factor that plays an important role in cell growth and differentiation, and its expression is restricted to a limited number of tissues, including gonads and kidney, as well as progenitor cells of various tissue types [4–7]. The protein is expressed as several isoforms. A major alternative splice donor site at the end of intron 9 results in the incorporation of three additional amino acids, lysine, threonine, and serine (KTS), between the third and fourth zinc fingers (Fig. 2a) [8]. The WT1 (–KTS) protein is believed to act primarily as a transcription factor, whereas the WT1 (+ KTS) protein is involved in post-transcriptional processes [9–12]. *WT1* variants in the germline are known to cause Wilms tumor as well as renal glomerulosclerosis and gonadal dysplasia.

Nephrotic syndrome (NS) is the most common glomerular disease in children and adults, characterized by massive proteinuria, hypoalbuminemia, and edema. Based on the response to glucocorticoid therapy, NS is classified into steroid-sensitive nephrotic syndrome or steroid-resistant nephrotic syndrome (SRNS). Focal segmental glomerulosclerosis (FSGS) is the most common histopathological finding in SRNS [13]. To date, more than 50 genes have been shown to cause monogenic SRNS and/or FSGS [14, 15]. Although detection rates vary by region and criteria, comprehensive genetic testing has identified podocyte-related gene variants in approximately 20%–30% of SRNS patients [14–18]. It has also been shown that there are differences in the distribution and frequency of variants by ethnic background and region. According to several studies conducted in Western countries, the three most common causative genes are *NPHS1*, *NPHS2*, and *WT1* [14, 16, 17]. In Asian countries, *WT1* is the most frequently detected causative gene [19–21]. Despite the high rate at which *WT1* is the causative gene in diseases that result in proteinuria, there are no definitive and detailed findings on the clinical manifestations of cases that do not meet the criteria for established syndromes such as Denys–Drash syndrome (DDS) and Frasier syndrome (FS). Actually, DDS has been asserted to be caused by missense variants in *WT1* only in exon 8–9 DNA binding sites and C2H2 sites [22]; however, many nephrologists still believe that many more variants including truncating variants in exons 8–9 can cause DDS.

Today, genetic testing is widely available and allows the risk stratification, management, and follow-up of patients with proteinuria. In order to treat patients with *WT1* variants, it is necessary to understand the possible symptoms depending on the location of the variant. In this review, we present the function and structure of the *WT1* gene, followed by a long-established and well-known syndrome to organize our knowledge and the association between its variants and clinical manifestations. And finally, we describe our recent findings on the correlation between genotype and phenotype, particularly in renal manifestations.

### Renal development

The kidneys develop from the intermediate mesoderm and are formed in three stages: anterior, middle, and posterior kidneys. Most of the anterior and middle kidneys later degenerate, and the kidneys that function in adult mammals are the posterior kidneys. In mammals, the kidney, or postrenal gland, arises from the most caudal part of the mesonephric duct (Wolff’s duct), in a process in which the ureteric bud emerges and mesenchyme assembles around it. The ureteric bud invades the metanephric mesenchyme and the metanephric mesenchyme cells condense around the ureteric bud tip to form the cap mesenchyme. The cap mesenchyme contains the nephrogenic progenitor cells that give rise to all epithelial cells of the nephron. The interaction of the ureteric bud with the posterior renal mesenchyme results in a posterior kidney with millions of nephrons. Mesenchymal cells aggregate around the ureteric bud, which epithelializes into an S-shaped body, giving rise to the glomerulus and the proximal and distal tubules. The ureteric bud undergoes a series of branching steps to become the collecting duct and ureter [23].

The earliest expression of Wt1 in the kidney was identified in the intermediate mesoderm and subsequently in the metanephric and cap mesenchyme [24, 25]. In the metanephric mesenchyme, Wt1 is required for the production of ureteric bud branching signals and for the nephrogenic progenitor cell response to ureteric bud-derived nephrogenic signals, demonstrating that this gene is crucial for the cell interactions during kidney formation [26, 27]. In later stages, Wt1 is involved in the control of mesenchymal–epithelial transition and has an essential role in the development and maintenance of podocytes [28–30].

### Structure

The Wilms tumor 1 (*WT1*) gene is located on chromosome 11p13 and encodes a transcriptional DNA binding protein. *WT1* contains 10 exons, which encode a proline/glutamine-rich transcriptional regulation region (exons 1–6) and four C2H2-zinc fingers (ZFs) (exons 7–10). The C2H2-zinc finger motif is employed by a diverse array of transcription factors that play important roles in cellular signal transduction [31] (Fig. 1).

Transcription factor plays an important role in cellular development and cell survival [4]. WT1 recognizes and binds to the DNA sequence 5′-GCG(T/G)GGGCG-3′ [31, 32] and regulates the expression of numerous target genes. WT1 also binds to the promoters and enhancers of several podocyte-specific genes [33–35].

The variants associated with DDS are predominantly missense variants in the first three ZFs, most often clustered in ZF2 and ZF3, that alter either the C2H2 structural amino acids that coordinate the zinc ions or the sequence-recognition amino acids at the protein–DNA interface (Fig. 1c, d). Because ZF2 and ZF3 correspond to exons 8 and 9, respectively, variants at these positions alter transcription factor activity and cause clinical symptoms.

There are at least 36 potential mammalian WT1 isoforms. This diversity is created through a combination of alternative transcription start sites, translation start sites, splicing, and RNA editing. Alternative splice sites at the end of exon 9 lead to the insertion of three amino acids (KTS) after the glycine between zinc fingers 3 and 4 [36]. This alternative splice site is highly conserved during evolution and is found in all vertebrates. The relative abundance of these splice forms is constant; developmental abnormalities are associated with altered ratios of WT1 (+ KTS) and WT1 (−KTS) isoforms with the standard ratio being almost 1:1 [37, 38] (Fig. 2). The + KTS and −KTS isoforms perform distinct biological functions and differ in their nucleic acid binding properties. The WT1 (−KTS) isoform binds DNA sequence-specifically and appears to function primarily in transcriptional regulation; more than 30 putative target genes of it have been identified. The WT1 (+ KTS) splice variant binds mRNA and plays a role in mRNA metabolism or splicing [39]. The unbalanced production of these isoforms causes FS.

### Clinical features and genetic background in *WT1*-related syndromes

WT1 is essential for normal urogenital development, and pathogenic variants in the *WT1* gene have been shown to be associated with syndromes such as DDS, FS, and Wilms–aniridia–genitourinary–mental retardation (WAGR) syndrome.

**DDS** (OMIM#194,080) is a disorder known to involve Wilms tumor, genital anomalies, and nephropathy. Its mode of inheritance is autosomal dominant. Familial cases have been reported, albeit rarely [40]. This syndrome was first described by Denys et al. in 1967 [41]. In 1970, Drash et al. reported two unrelated children with a syndrome comprising pseudohermaphroditism, Wilms tumor, hypertension, and degenerative renal disease [42]. In 10 independent cases of DDS, missense variants in the C2H2-zinc finger domains of one *WT1* gene allele were found in 1991 [43]. Nine of these variants were found within exon 9 (C2H2-zinc finger 3), while the tenth was in exon 8 (C2H2-zinc finger 2). These variants directly affect DNA sequence recognition, that is, DNA binding ability. A small number of cases of DDS associated with variants other than those in exons 8 and 9 have been reported [44, 45]. Lipska et al. presented clinical information on 24 patients with missense variants in DNA binding sites. The median age of onset was 0.9 and the median age of developing ESKD was 2.5 years. Other clinical manifestations included pathological findings of diffuse mesangial sclerosis (DMS) in 74% (17/23), Wilms tumor in 54% (13/24), genital abnormalities in 43% (9/21), and urinary tract malformation in 4% (1/24) [46]. Heterozygous *WT1* missense variants (mainly zinc finger domains) in DDS lead to a severe phenotype compared with deletions in WAGR syndrome [25]. The mutant WT1 protein dimerizes with the wild-type protein and acts in a dominant-negative manner, which may explain the more severe phenotype compared with haploinsufficiency [47].

**FS** (OMIM#136,680) is a disorder known to involve genital anomalies and progressive glomerulopathy. FS results from variants that reduce the + KTS to −KTS ratio by disrupting the splice donor of the + KTS isoform (Fig. 2b). Developing Wilms tumor in FS is less common than DDS. Its mode of inheritance is autosomal dominant and familial cases of FS have been reported [48]. In 1987, Moorthy et al. suggested that some of the patients reported to have DDS in fact had a different disorder, for which they suggested the designation “FS” [49]. The first confirmed case of FS was reported by Frasier et al. in 1964 [50]. In 1997, Barbaux et al. identified variants in the donor splice site of intron 9 of the *WT1* gene with a predicted loss of the so-called + KTS isoform in three patients with FS [51]. Subsequently, Lipska et al. revealed clinical information on 19 patients with intron 9 variants. In this group, unlike in patients with DNA binding site variants, the pathological findings were focal segmental glomerulosclerosis in 88% (15/17) and DMS in 6% (1/17) [46].

**WAGR syndrome** (OMIM#194,072) is a disorder known to involve Wilms tumor, aniridia, genital anomalies, and impaired intellectual development syndrome. In 1964, Miller et al. first described the association of aniridia, hemihypertrophy, and other congenital anomalies with Wilms tumor [52]. In 1988, Puissant et al. reported a patient with WAGR and a de novo reciprocal translocation, 46,XY,t(5;11)(q11;p13) [53]. WAGR syndrome is a contiguous gene deletion syndrome involving an interstitial de novo 11p13 microdeletion of variable size, which explains the variability of clinical signs [54]. The affected genes include *PAX6* and *WT1*. *BDNF* deletion can be included among the causative variants and is associated with the WAGRO phenotype (OMIM# 612,469). WAGRO is a specific phenotype of WAGR, with the additional feature of obesity, and is associated with haploinsufficiency of the *BDNF* gene.

### Clinical features and genetic background in *WT1*-related disorders

#### CAKUT (congenital anomalies of the kidney and urinary tract) phenotype

WT1 is required for ureter induction, formation of the nephron, and differentiation and maintenance of the glomerulus. Mice carrying *Wt1* variants lack kidney, exhibit renal dysplasia, or develop renal failure [55]. It was reported that 11% (7/61) of patients with a *WT1* variant had CAKUT, including duplex kidney, horseshoe kidney, malrotation, vesicoureteral reflex, and pelviureteric junction stenosis [46]. Three of the patients had intron 9 variants, two had truncation variants, and the rest had one DNA binding site variant and one other missense variant each.

#### Wilms tumor

Wilms tumor is the most common renal malignancy in pediatric populations. Approximately 9%–17% of all Wilms tumors are associated with a predisposing syndrome [56], the most common of which are WAGR, DDS, Beckwith–Wiedemann syndrome, isolated hemihypertrophy, and Perlman syndrome [57]. The risk of developing malignancy varies by syndrome. WAGR and DDS are classified as at high risk (> 20%) of developing Wilms tumor, while Frasier syndrome is classified as at moderate risk (5%–20%).

According to a review of 150 cases published in 1994, patients with DDS have a likelihood of developing Wilms tumor as high as 95%, with median age at occurrence of 12 months [58]. Among children with DDS and Wilms tumor, 20% have bilateral masses [58, 59].

Lipska et al. reported that Wilms tumors developed in 23 out of 61 patients with *WT1*-related steroid-resistant nephrotic syndrome. Of them, 22 were carriers of exonic *WT1* variants who were cumulatively followed up for 199 years (that is, one case per 9 years at risk). Overall, 13 of 24 (54%) with missense variants, 7 of 9 (78%) with truncating variants, 2 of 7 (29%) with other missense variants, and 1 of 19 (5%) with intron 9 variants developed Wilms tumor.

Lehnhardt et al. reported that only patients with exon variants developed Wilms tumor [60]. A total of 12 of 36 (33%) with missense variants and 7 of 8 (88%) with truncating variants developed Wilms tumor [60].

We reported that 36% (47/129 cases) of patients with exon 8–9 *WT1* variants had Wilms tumor. This included 24 of 69 cases (35%) in the DNA binding site (DBS) group, 17 of 32 (53%) in the C2H2 group, and 6 of 28 (21%) in the Others group. Our group also reviewed 126 cases with *WT1* intron 9 variants, in which the prevalence of Wilms tumor was 3% (1/30 cases) [38]. Overall, patients with truncating variants developed Wilms tumor at a high frequency, and patients with missense variants at any position, albeit quite rarely for those with missense variants at locations other than exon 8 or 9, were at risk of developing Wilms tumor.

#### Gonadal development

*WT1* is one of the key genes involved in the development of the gonads and adrenal glands [61]. According to Köhler et al. [62], patients with 46,XY disorders of sex development (DSD) with *WT1* variants identified by histological analysis of gonadal tissue showed a large spectrum of development, ranging from normal testes to varying degrees of gonadal dysgenesis, but patients with *WT1* pathogenic variants lacked a clear genotype–phenotype correlation.

Among patients with truncating pathogenic variants, which are nonsense, frameshift, and splice site variants other than intron 9 variants, the majority of 46,XY patients often have genital dysgenesis (46,XY DSD) and fewer have normal female external and internal genitalia (46,XY complete gonadal dysgenesis) [60]. Among patients with missense variants in exon 8 or 9 with or without DBS, the majority of 46,XY patients have genital anomalies/atypia (46,XY DSD), and 46,XY complete gonadal dysgenesis is rare [63]. Among patients with intron 9 variants, the diagnosis of 46,XY complete gonadal dysgenesis is observed in the majority of 46,XY patients, but partial forms also occur [60, 64].

#### Gonadoblastoma

A gonadoblastoma is a complex neoplasm composed of a mixture of gonadal elements, such as large primordial germ cells, immature Sertoli cells or granulosa cells of the sex cord, and gonadal stromal cells [65]. Classical gonadoblastoma occurs almost entirely in the dysgenetic gonads of individuals with disorders of sex development; however, a small number of cases arise in individuals with a normal peripheral karyotype and no evidence of a disorder of sex development [66]. Even in normal external male genitalia, testicular hypoplasia or dysplasia is possible and may lead to gonadoblastoma. Therefore, periodic examination and testing are necessary.

Gonadoblastoma has been identified in both FS and DDS, but the risk of it in FS is much higher than that in DDS [67]. However, the risk of gonadoblastoma should not be overlooked even in patients with DDS.

### Deeper insights into genotype–phenotype correlations

#### Missense variants in exon 8 or 9

Based on crystallographic analysis [31], there are two types of disease-causing variants in exon8 or 9, which either destabilize the zinc finger structure or replace important base contact residues. We focused on the structure and classified the variants into three categories: DNA binding sites, C2H2 sites, and other sites. Genotype–phenotype correlations were evaluated in a systematic review of 174 cases with *WT1* exon 8 to 9 variants [22]. There were 95 DNA binding site variants, 38 C2H2 site variants, and 41 other site variants. The median age of developing ESKD was 0.90 in the DNA binding site group, 2.00 in the C2H2 site group, and 3.92 years in the other site group. We concluded that not only DNA binding sites but also C2H2 zinc finger structure sites are important for maintaining *WT1* transcriptional activity, and their mutation causes severe clinical symptoms (Table 1).

**Table 1:** Genotype–phenotype association for *WT1*-related disorders

Type of variant		Exonic					Intronic	Gene
		missense variant				truncating variant	intron 9 variant	whole deletion
		exon8 or 9			Other than exon8 and 9			
		DNA binding site	C2H2 site	other				
nephropathy	time of onset	newborn-infancy	infancy	infancy	variable	(if it occurs) middle childhood	early childhood	(if it occurs) middle childhood
	ESKD	infancy	infancy-early childhood	early childhood	variable	(if it occurs) adolescence	adolescence	(if it occurs) adolescence
Wilms tumor		moderate			rare	high	rare	high
Genital abnormalities		moderate				moderate	moderate	high
Gonadoblastoma		rare				unknown	high	high

Recently, severe cases of fetal onset and early neonatal death due to *WT1* variants have been reported [68]. These patients have a heterozygous missense variant in *WT1* [NM_024426.6:exon9:c.1400G > A, p.Arg467Gln]. Five cases with the same missense variant were reported. The median age of onset was 0.08 years and the median age of ESKD was 0.2 years, which is extremely severe even for DDS in this genotype. The most common variant in DDS involves the same amino acid but with it changing to tryptophan (p.Arg467Trp). Different amino acid changes in the same DNA binding site may affect transcriptional activity.

#### Intron 9 variants

Genotype–phenotype correlations were evaluated in a systematic review of 126 cases with *WT1* intron 9 variants [38]. Patients included 3 with +1G > A, 2 with +2 T > C, 1 with 3 + G > T, 66 with +4C > T, 51 with +5G > A, 2 with +5G > T, and 1 with +6 T > A. The median age of onset of proteinuria was 4 years. Furthermore, the median age of developing ESKD was 16 years. There were no significant differences in the renal survival period among the genotypes.

#### Missense variants other than in exon 8 or 9

Because missense variants in *WT1* at locations other than exon 8 or 9 are so rare, no comprehensive reports on such cases have been published and the details remain unclear. At the time of writing, 28 pathogenic variants at locations other than exon 8 or 9 that are associated with nephropathy have been reported in the Human Gene Mutation Database (HGMD) Professional v2024.1 (https://portal.biobase-international.com/hgmd/pro/start.php). Their locations are as follows: 9 variants in exon 1, 3 variants in exon 2, 1 variant in exon 4, 2 variants in exon 6, 12 variants in exon 7, and 1 variant in exon 10. In many cases, the details of clinical symptoms are unknown, but the severity is known to vary.

#### Truncating variants

A genotype–phenotype association study in *WT1*-related disorders with a large number of cases was reported in 2014 [46]. In that study, 61 patients with *WT1*-related steroid-resistant nephrotic syndrome in the PodoNet cohort were evaluated. Seven truncating variants were identified. The median age of onset of nephropathy was 12.3 years and the median age of developing ESKD was 16.5 years.

Meanwhile, in 2015, a retrospective genotypic, phenotypic, and therapeutic analysis of 53 patients with *WT1* variants from Germany, Austria, and Switzerland was performed [60]. A total of 8 of 53 (15%) patients had a truncating variant. The median age of onset of nephropathy was 9.7 years, 73% of patients required RRT, and the median age of developing ESKD was 16.5 years.

In both cohorts, cases presenting with nephropathy were collected. In fact, 31 truncating variants have been reported in HGMD v2024.1 to date, 22 of which were associated with clinical manifestations of Wilms tumor only. The presence of a truncating variant is associated with a higher rate of Wilms tumor than nephropathy. Since all variants are located before the DNA binding site of exon 9, the renal symptoms may be milder because dominant-negative effects on DNA binding may be rare.

### Future challenges

Patients with renal symptoms who have missense variants in exons 8 or 9 and patients with Wilms tumor who have truncating variants are relatively well reported. However, there are still few reports of patients with variants in other positions, and a clear genotype–phenotype correlations have not been established. Since genetic testing is now easily available, it is expected that more and more patients will be found with only one clinical symptom. It is necessary to follow such patients over a long period and carefully monitor their progress to accumulate data on the types of clinical symptoms they present. Future work will consider the functional analysis of these data to elucidate the mechanisms by which pathological variants in the *WT1* gene cause their respective symptoms. We believe that such research will eventually lead to gene therapy.

## Conclusion

As genetic testing proliferates, an increasing number of patients with *WT1* variants are being identified. It has become clear that discrepancies in symptom manifestation in kidney disorders arise based on the variant’s location. Beyond renal complications, urogenital anomalies and tumorigenesis can occur, underscoring the importance of a wider awareness of such cases as *WT1*-related disorders beyond the field of nephrology. Against this background, recent progress has deepened our understanding of genotype–phenotype correlations in *WT1*-related disorders.

## References

[1] Call KM, Glaser T, Ito CY, Buckler AJ, Pelletier J, Haber DA, et al. Isolation and characterization of a zinc finger polypeptide gene at the human chromosome 11 Wilms’ tumor locus. Cell. 1990;60(3):509–20. 10.1016/0092-8674(90)90601-a. 2154335 10.1016/0092-8674(90)90601-a

[2] Rose EA, Glaser T, Jones C, Smith CL, Lewis WH, Call KM, et al. Complete physical map of the WAGR region of 11p13 localizes a candidate Wilms’ tumor gene. Cell. 1990;60(3):495–508. 10.1016/0092-8674(90)90600-j. 2154334 10.1016/0092-8674(90)90600-j

[3] Morris JF, Madden SL, Tournay OE, Cook DM, Sukhatme VP, Rauscher FJ 3rd. Characterization of the zinc finger protein encoded by the WT1 Wilms’ tumor locus. Oncogene. 1991;6(12):2339–48. 1662794

[4] Hamilton TB, Barilla KC, Romaniuk PJ. High affinity binding sites for the Wilms’ tumour suppressor protein WT1. Nucleic Acids Res. 1995;23(2):277–84. 10.1093/nar/23.2.277. 7862533 10.1093/nar/23.2.277 PMC306666

[5] Buckler AJ, Pelletier J, Haber DA, Glaser T, Housman DE. Isolation, characterization, and expression of the murine Wilms’ tumor gene (WT1) during kidney development. Mol Cell Biol. 1991;11(3):1707–12. 10.1128/mcb.11.3.1707-1712.1991. 1671709 10.1128/mcb.11.3.1707-1712.1991 PMC369476

[6] Park S, Schalling M, Bernard A, Maheswaran S, Shipley GC, Roberts D, et al. The Wilms tumour gene WT1 is expressed in murine mesoderm-derived tissues and mutated in a human mesothelioma. Nat Genet. 1993;4(4):415–20. 10.1038/ng0893-415. 8401592 10.1038/ng0893-415

[7] Davies R, Moore A, Schedl A, Bratt E, Miyahawa K, Ladomery M et al. Multiple roles for the Wilms' tumor suppressor, WT1. Cancer Res. 1999;59(7 Suppl):1747s-50s; discussion 51s. 10197591

[8] Haber DA, Sohn RL, Buckler AJ, Pelletier J, Call KM, Housman DE. Alternative splicing and genomic structure of the Wilms tumor gene WT1. Proc Natl Acad Sci U S A. 1991;88(21):9618–22. 10.1073/pnas.88.21.9618. 1658787 10.1073/pnas.88.21.9618 PMC52769

[9] Larsson SH, Charlieu JP, Miyagawa K, Engelkamp D, Rassoulzadegan M, Ross A, et al. Subnuclear localization of WT1 in splicing or transcription factor domains is regulated by alternative splicing. Cell. 1995;81(3):391–401. 10.1016/0092-8674(95)90392-5. 7736591 10.1016/0092-8674(95)90392-5

[10] Caricasole A, Duarte A, Larsson SH, Hastie ND, Little M, Holmes G, et al. RNA binding by the Wilms tumor suppressor zinc finger proteins. Proc Natl Acad Sci U S A. 1996;93(15):7562–6. 10.1073/pnas.93.15.7562. 8755514 10.1073/pnas.93.15.7562 PMC38785

[11] Niksic M, Slight J, Sanford JR, Caceres JF, Hastie ND. The Wilms’ tumour protein (WT1) shuttles between nucleus and cytoplasm and is present in functional polysomes. Hum Mol Genet. 2004;13(4):463–71. 10.1093/hmg/ddh040. 14681305 10.1093/hmg/ddh040

[12] Bor YC, Swartz J, Morrison A, Rekosh D, Ladomery M, Hammarskjold ML. The Wilms’ tumor 1 (WT1) gene (+KTS isoform) functions with a CTE to enhance translation from an unspliced RNA with a retained intron. Genes Dev. 2006;20(12):1597–608. 10.1101/gad.1402306. 16738405 10.1101/gad.1402306 PMC1482480

[13] Warejko JK, Tan W, Daga A, Schapiro D, Lawson JA, Shril S, et al. Whole Exome Sequencing of Patients with Steroid-Resistant Nephrotic Syndrome. Clin J Am Soc Nephrol. 2018;13(1):53–62. 10.2215/CJN.04120417. 29127259 10.2215/CJN.04120417 PMC5753307

[14] Bierzynska A, McCarthy HJ, Soderquest K, Sen ES, Colby E, Ding WY, et al. Genomic and clinical profiling of a national nephrotic syndrome cohort advocates a precision medicine approach to disease management. Kidney Int. 2017;91(4):937–47. 10.1016/j.kint.2016.10.013. 28117080 10.1016/j.kint.2016.10.013

[15] Preston R, Stuart HM, Lennon R. Genetic testing in steroid-resistant nephrotic syndrome: why, who, when and how? Pediatr Nephrol. 2017. 10.1007/s00467-017-3838-6. 29181713 10.1007/s00467-017-3838-6 PMC6311200

[16] Sadowski CE, Lovric S, Ashraf S, Pabst WL, Gee HY, Kohl S et al. A single-gene cause in 29.5% of cases of steroid-resistant nephrotic syndrome. J Am Soc Nephrol. 2015;26(6):1279–89. 10.1681/ASN.2014050489. 10.1681/ASN.2014050489 PMC4446877 25349199

[17] Trautmann A, Lipska-Zietkiewicz BS, Schaefer F. Exploring the clinical and genetic spectrum of steroid resistant nephrotic syndrome: The PodoNet Registry. Front Pediatr. 2018;6:200. 10.3389/fped.2018.00200. 30065916 10.3389/fped.2018.00200 PMC6057105

[18] Varner JD, Chryst-Stangl M, Esezobor CI, Solarin A, Wu G, Lane B, et al. Genetic testing for steroid-resistant-nephrotic syndrome in an outbred population. Front Pediatr. 2018;6:307. 10.3389/fped.2018.00307. 30406062 10.3389/fped.2018.00307 PMC6204400

[19] Nagano C, Yamamura T, Horinouchi T, Aoto Y, Ishiko S, Sakakibara N, et al. Comprehensive genetic diagnosis of Japanese patients with severe proteinuria. Sci Rep. 2020;10(1):270. 10.1038/s41598-019-57149-5. 31937884 10.1038/s41598-019-57149-5 PMC6959278

[20] Park E, Lee C, Kim NKD, Ahn YH, Park YS, Lee JH et al. Genetic study in Korean pediatric patients with steroid-resistant nephrotic syndrome or focal segmental glomerulosclerosis. J Clin Med. 2020;9(6). 10.3390/jcm9062013. 10.3390/jcm9062013 PMC7355646 32604935

[21] Zhang L, Zhao F, Ding G, Chen Y, Zhao S, Chen Q et al. Monogenic causes identified in 23.68% of children with steroid-resistant nephrotic syndrome: a single-centre study. Kidney Dis. 2024;10(1):61–8. 10.1159/000534853. 10.1159/000534853 PMC10843177 38322629

[22] Nagano C, Takaoka Y, Kamei K, Hamada R, Ichikawa D, Tanaka K, et al. Genotype-phenotype correlation in WT1 Exon 8 to 9 missense variants. Kidney Int Rep. 2021;6(8):2114–21. 10.1016/j.ekir.2021.05.009. 34386660 10.1016/j.ekir.2021.05.009 PMC8343804

[23] Little MH, McMahon AP. Mammalian kidney development: principles, progress, and projections. Cold Spring Harb Perspect Biol. 2012;4(5). 10.1101/cshperspect.a008300. 10.1101/cshperspect.a008300 PMC3331696 22550230

[24] Pritchard-Jones K, Fleming S, Davidson D, Bickmore W, Porteous D, Gosden C, et al. The candidate Wilms’ tumour gene is involved in genitourinary development. Nature. 1990;346(6280):194–7. 10.1038/346194a0. 2164159 10.1038/346194a0

[25] Pelletier J, Schalling M, Buckler AJ, Rogers A, Haber DA, Housman D. Expression of the Wilms’ tumor gene WT1 in the murine urogenital system. Genes Dev. 1991;5(8):1345–56. 10.1101/gad.5.8.1345. 1651275 10.1101/gad.5.8.1345

[26] Kreidberg JA, Sariola H, Loring JM, Maeda M, Pelletier J, Housman D, et al. WT-1 is required for early kidney development. Cell. 1993;74(4):679–91. 10.1016/0092-8674(93)90515-r. 8395349 10.1016/0092-8674(93)90515-r

[27] Donovan MJ, Natoli TA, Sainio K, Amstutz A, Jaenisch R, Sariola H, et al. Initial differentiation of the metanephric mesenchyme is independent of WT1 and the ureteric bud. Dev Genet. 1999;24(3–4):252–62. 10.1002/(SICI)1520-6408(1999)24:3/4%3c252::AID-DVG8%3e3.0.CO;2-K. 10322633 10.1002/(SICI)1520-6408(1999)24:3/4<252::AID-DVG8>3.0.CO;2-K

[28] Davies JA, Ladomery M, Hohenstein P, Michael L, Shafe A, Spraggon L, et al. Development of an siRNA-based method for repressing specific genes in renal organ culture and its use to show that the Wt1 tumour suppressor is required for nephron differentiation. Hum Mol Genet. 2004;13(2):235–46. 10.1093/hmg/ddh015. 14645201 10.1093/hmg/ddh015

[29] Morrison AA, Viney RL, Saleem MA, Ladomery MR. New insights into the function of the Wilms tumor suppressor gene WT1 in podocytes. Am J Physiol Renal Physiol. 2008;295(1):F12–7. 10.1152/ajprenal.00597.2007. 18385267 10.1152/ajprenal.00597.2007

[30] Miller-Hodges E, Hohenstein P. WT1 in disease: shifting the epithelial-mesenchymal balance. J Pathol. 2012;226(2):229–40. 10.1002/path.2977. 21959952 10.1002/path.2977

[31] Stoll R, Lee BM, Debler EW, Laity JH, Wilson IA, Dyson HJ, et al. Structure of the Wilms tumor suppressor protein zinc finger domain bound to DNA. J Mol Biol. 2007;372(5):1227–45. 10.1016/j.jmb.2007.07.017. 17716689 10.1016/j.jmb.2007.07.017

[32] Hashimoto H, Olanrewaju YO, Zheng Y, Wilson GG, Zhang X, Cheng X. Wilms tumor protein recognizes 5-carboxylcytosine within a specific DNA sequence. Genes Dev. 2014;28(20):2304–13. 10.1101/gad.250746.114. 25258363 10.1101/gad.250746.114 PMC4201290

[33] Dong L, Pietsch S, Englert C. Towards an understanding of kidney diseases associated with WT1 mutations. Kidney Int. 2015;88(4):684–90. 10.1038/ki.2015.198. 26154924 10.1038/ki.2015.198 PMC4687464

[34] Kann M, Bae E, Lenz MO, Li L, Trannguyen B, Schumacher VA, et al. WT1 targets Gas1 to maintain nephron progenitor cells by modulating FGF signals. Development. 2015;142(7):1254–66. 10.1242/dev.119735. 25804736 10.1242/dev.119735 PMC4378252

[35] Lefebvre J, Clarkson M, Massa F, Bradford ST, Charlet A, Buske F, et al. Alternatively spliced isoforms of WT1 control podocyte-specific gene expression. Kidney Int. 2015;88(2):321–31. 10.1038/ki.2015.140. 25993318 10.1038/ki.2015.140

[36] Wuttke DS, Foster MP, Case DA, Gottesfeld JM, Wright PE. Solution structure of the first three zinc fingers of TFIIIA bound to the cognate DNA sequence: determinants of affinity and sequence specificity. J Mol Biol. 1997;273(1):183–206. 10.1006/jmbi.1997.1291. 9367756 10.1006/jmbi.1997.1291

[37] Elrod-Erickson M, Benson TE, Pabo CO. High-resolution structures of variant Zif268-DNA complexes: implications for understanding zinc finger-DNA recognition. Structure. 1998;6(4):451–64. 10.1016/s0969-2126(98)00047-1. 9562555 10.1016/s0969-2126(98)00047-1

[38] Tsuji Y, Yamamura T, Nagano C, Horinouchi T, Sakakibara N, Ishiko S, et al. Systematic review of genotype-phenotype correlations in frasier syndrome. Kidney Int Rep. 2021;6(10):2585–93. 10.1016/j.ekir.2021.07.010. 34622098 10.1016/j.ekir.2021.07.010 PMC8484119

[39] Markus MA, Heinrich B, Raitskin O, Adams DJ, Mangs H, Goy C, et al. WT1 interacts with the splicing protein RBM4 and regulates its ability to modulate alternative splicing in vivo. Exp Cell Res. 2006;312(17):3379–88. 10.1016/j.yexcr.2006.07.008. 16934801 10.1016/j.yexcr.2006.07.008

[40] Royer-Pokora B, Beier M, Henzler M, Alam R, Schumacher V, Weirich A, et al. Twenty-four new cases of WT1 germline mutations and review of the literature: genotype/phenotype correlations for Wilms tumor development. Am J Med Genet A. 2004;127A(3):249–57. 10.1002/ajmg.a.30015. 15150775 10.1002/ajmg.a.30015

[41] Denys P, Malvaux P, Van Den Berghe H, Tanghe W, Proesmans W. Association of an anatomo-pathological syndrome of male pseudohermaphroditism, Wilms’ tumor, parenchymatous nephropathy and XX/XY mosaicism. Arch Fr Pediatr. 1967;24(7):729–39. 4292870

[42] Drash A, Sherman F, Hartmann WH, Blizzard RM. A syndrome of pseudohermaphroditism, Wilms’ tumor, hypertension, and degenerative renal disease. J Pediatr. 1970;76(4):585–93. 10.1016/s0022-3476(70)80409-7. 4316066 10.1016/s0022-3476(70)80409-7

[43] Pelletier J, Bruening W, Kashtan CE, Mauer SM, Manivel JC, Striegel JE, et al. Germline mutations in the Wilms’ tumor suppressor gene are associated with abnormal urogenital development in Denys-Drash syndrome. Cell. 1991;67(2):437–47. 10.1016/0092-8674(91)90194-4. 1655284 10.1016/0092-8674(91)90194-4

[44] Bruening W, Bardeesy N, Silverman BL, Cohn RA, Machin GA, Aronson AJ, et al. Germline intronic and exonic mutations in the Wilms’ tumour gene (WT1) affecting urogenital development. Nat Genet. 1992;1(2):144–8. 10.1038/ng0592-144. 1302008 10.1038/ng0592-144

[45] McCoy FE Jr, Franklin WA, Aronson AJ, Spargo BH. Glomerulonephritis associated with male pseudohermaphroditism and nephroblastoma. Am J Surg Pathol. 1983;7(4):387–95. 10.1097/00000478-198306000-00011. 6307071 10.1097/00000478-198306000-00011

[46] Lipska BS, Ranchin B, Iatropoulos P, Gellermann J, Melk A, Ozaltin F, et al. Genotype-phenotype associations in WT1 glomerulopathy. Kidney Int. 2014;85(5):1169–78. 10.1038/ki.2013.519. 24402088 10.1038/ki.2013.519

[47] Little M, Holmes G, Bickmore W, van Heyningen V, Hastie N, Wainwright B. DNA binding capacity of the WT1 protein is abolished by Denys-Drash syndrome WT1 point mutations. Hum Mol Genet. 1995;4(3):351–8. 10.1093/hmg/4.3.351. 7795587 10.1093/hmg/4.3.351

[48] Klamt B, Koziell A, Poulat F, Wieacker P, Scambler P, Berta P, et al. Frasier syndrome is caused by defective alternative splicing of WT1 leading to an altered ratio of WT1 +/-KTS splice isoforms. Hum Mol Genet. 1998;7(4):709–14. 10.1093/hmg/7.4.709. 9499425 10.1093/hmg/7.4.709

[49] Moorthy AV, Chesney RW, Lubinsky M. Chronic renal failure and XY gonadal dysgenesis: “Frasier” syndrome–a commentary on reported cases. Am J Med Genet Suppl. 1987;3:297–302. 10.1002/ajmg.1320280535. 3130865 10.1002/ajmg.1320280535

[50] Frasier SD, Bashore RA, Mosier HD. Gonadoblastoma associated with pure gonadal dysgenesis in monozygous twins. J Pediatr. 1964;64:740–5. 10.1016/s0022-3476(64)80622-3. 14149008 10.1016/s0022-3476(64)80622-3

[51] Barbaux S, Niaudet P, Gubler MC, Grunfeld JP, Jaubert F, Kuttenn F, et al. Donor splice-site mutations in WT1 are responsible for Frasier syndrome. Nat Genet. 1997;17(4):467–70. 10.1038/ng1297-467. 9398852 10.1038/ng1297-467

[52] Miller RW, Fraumeni JF Jr, Manning MD. Association of wilms’s tumor with aniridia, hemihypertrophy and other congenital malformations. N Engl J Med. 1964;270:922–7. 10.1056/NEJM196404302701802. 14114111 10.1056/NEJM196404302701802

[53] Puissant H, Azoulay M, Serre JL, Piet LL, Junien C. Molecular analysis of a reciprocal translocation t(5;11) (q11;p13) in a WAGR patient. Hum Genet. 1988;79(3):280–2. 10.1007/BF00366252. 2841227 10.1007/BF00366252

[54] Iijima K, Someya T, Ito S, Nozu K, Nakanishi K, Matsuoka K, et al. Focal segmental glomerulosclerosis in patients with complete deletion of one WT1 allele. Pediatrics. 2012;129(6):e1621–5. 10.1542/peds.2011-1323. 22585769 10.1542/peds.2011-1323

[55] Schedl A. Renal abnormalities and their developmental origin. Nat Rev Genet. 2007;8(10):791–802. 10.1038/nrg2205. 17878895 10.1038/nrg2205

[56] Segers H, Kersseboom R, Alders M, Pieters R, Wagner A, van den Heuvel-Eibrink MM. Frequency of WT1 and 11p15 constitutional aberrations and phenotypic correlation in childhood Wilms tumour patients. Eur J Cancer. 2012;48(17):3249–56. 10.1016/j.ejca.2012.06.008. 22796116 10.1016/j.ejca.2012.06.008

[57] Scott RH, Stiller CA, Walker L, Rahman N. Syndromes and constitutional chromosomal abnormalities associated with Wilms tumour. J Med Genet. 2006;43(9):705–15. 10.1136/jmg.2006.041723. 16690728 10.1136/jmg.2006.041723 PMC2564568

[58] Mueller RF. The Denys-Drash syndrome. J Med Genet. 1994;31(6):471–7. 10.1136/jmg.31.6.471. 8071974 10.1136/jmg.31.6.471 PMC1049926

[59] Charlton J, Irtan S, Bergeron C, Pritchard-Jones K. Bilateral Wilms tumour: a review of clinical and molecular features. Expert Rev Mol Med. 2017;19: e8. 10.1017/erm.2017.8. 28716159 10.1017/erm.2017.8 PMC5687181

[60] Lehnhardt A, Karnatz C, Ahlenstiel-Grunow T, Benz K, Benz MR, Budde K, et al. Clinical and molecular characterization of patients with heterozygous mutations in wilms tumor suppressor gene 1. Clin J Am Soc Nephrol. 2015;10(5):825–31. 10.2215/CJN.10141014. 25818337 10.2215/CJN.10141014 PMC4422247

[61] Bandiera R, Sacco S, Vidal VP, Chaboissier MC, Schedl A. Steroidogenic organ development and homeostasis: A WT1-centric view. Mol Cell Endocrinol. 2015;408:145–55. 10.1016/j.mce.2015.01.009. 25596547 10.1016/j.mce.2015.01.009

[62] Kohler B, Biebermann H, Friedsam V, Gellermann J, Maier RF, Pohl M, et al. Analysis of the Wilms’ tumor suppressor gene (WT1) in patients 46, XY disorders of sex development. J Clin Endocrinol Metab. 2011;96(7):E1131–6. 10.1210/jc.2010-2804. 21508141 10.1210/jc.2010-2804

[63] Lipska-Zietkiewicz BS. WT1 Disorder. In: Adam MP, Feldman J, Mirzaa GM, Pagon RA, Wallace SE, Bean LJH et al., editors. GeneReviews((R)). Seattle (WA)1993. 32352694

[64] Chernin G, Vega-Warner V, Schoeb DS, Heeringa SF, Ovunc B, Saisawat P, et al. Genotype/phenotype correlation in nephrotic syndrome caused by WT1 mutations. Clin J Am Soc Nephrol. 2010;5(9):1655–62. 10.2215/CJN.09351209. 20595692 10.2215/CJN.09351209 PMC2974408

[65] Cools M, Stoop H, Kersemaekers AM, Drop SL, Wolffenbuttel KP, Bourguignon JP, et al. Gonadoblastoma arising in undifferentiated gonadal tissue within dysgenetic gonads. J Clin Endocrinol Metab. 2006;91(6):2404–13. 10.1210/jc.2005-2554. 16608895 10.1210/jc.2005-2554

[66] Roth LM, Cheng L. Gonadoblastoma: origin and outcome. Hum Pathol. 2020;100:47–53. 10.1016/j.humpath.2019.11.005. 31805291 10.1016/j.humpath.2019.11.005

[67] Patel PR, Pappas J, Arva NC, Franklin B, Brar PC. Early presentation of bilateral gonadoblastomas in a Denys-Drash syndrome patient: a cautionary tale for prophylactic gonadectomy. J Pediatr Endocrinol Metab. 2013;26(9–10):971–4. 10.1515/jpem-2012-0409. 23729537 10.1515/jpem-2012-0409

[68] Yoshino M, Shimabukuro W, Takeichi M, Omura J, Yokota C, Yamamoto J, et al. A case of Potter sequence with WT1 mutation. CEN Case Rep. 2023;12(2):184–8. 10.1007/s13730-022-00742-x. 36227513 10.1007/s13730-022-00742-x PMC10151295

